# Short report on implications of Covid-19 and emerging zoonotic infectious diseases for pastoralists and Africa

**DOI:** 10.1186/s13570-020-00173-2

**Published:** 2020-06-09

**Authors:** Anthony Egeru, Sintayehu W. Dejene, Aggrey Siya

**Affiliations:** 1 Department of Environmental Management, College of Agricultural and Environmental Science, Makerere University, P.O. Box 7062, Kampala, Uganda; 2 Training and Community Development, Regional Universities Forum for Capacity Building in Agriculture, P.O. Box 16811, Wandegeya, Kampala, Uganda; 3 College of Agriculture and Environmental Sciences, Haramaya University, P.O. Box 282, Dire Dawa, Ethiopia; 4 College of Veterinary Medicine, Animal Resources and Biosecurity, Makerere University, P.O. Box 7062, Kampala, Uganda

**Keywords:** Epidemics, Emergence, Livestock, MERS-CoV, One Health, Pandemic, SARS-CoV-2

## Abstract

Many emerging and re-emerging zoonotic infectious diseases occur in Africa. These are projected to increase as human–animal host contact increases owing to increasing environmental degradation that shrinks nature habitats for wildlife over the continent. The current outbreak of severe acute respiratory syndrome coronavirus-2 (SARS-CoV-2) responsible for causing coronavirus disease in 2019 (COVID-19) has reinvigorated discourse on the disruptiveness of the zoonotic emerging infectious diseases, owing to their transboundary character. Even as the world focuses on the COVID-19 sweeping pandemic, the Middle East respiratory syndrome coronavirus (MERS)-CoV re-emerged in Saudi Arabia infecting 18 people with five deaths; this has barely received any attention. This outbreak is particularly of concern to the pastoralists in the Horn of Africa, a region that has in recent past seen an increase in camel trade with the Gulf States, especially Yemen and Saudi Arabia. Emerging and re-emerging zoonotic infectious diseases are complex, depend on human–animal–environment interaction and pose a strain on public health systems. There is a need to address these diseases dynamically through a synergistic approach, drawing on expertise from diverse sectors. One Health approach has distinguished itself as an integrative action able to bring together multiple actors on a global, national and local scale to advance the attainment of optimal health outcomes for people, animals and the environment. One Health works by strengthening the preparedness, response, mitigation and monitoring of zoonotic infectious disease risks collaboratively. We opine that as zoonotic emerging and re-emerging infectious diseases continue to rise over pastoral Africa, comprehensive implementation of the One Health approach will be urgently required.

## Introduction

Emergence and re-emergence of most infectious diseases affecting humans have been linked to viruses originating from zoonotic transmission through human-animal host contact (Mollentze and Streicker 2020). Zoonotic infectious diseases are thus diseases that are transmitted between animals and people (Sack et al. 2018). Human–animal (host) contact in most pastoral communities in Africa is diverse, primarily because of the diversity of animals as well as viruses in nature; these lead to a diversity of zoonotic infectious diseases’ prevalence. Pastoral communities are at a high risk of interacting with zoonotic infectious diseases, owing to their livestock management practices, which include herd mixing and transhumance and the locations they occupy - especially their contact with wildlife - as well as the consumption of bushmeat among some pastoral communities (Ceppi and Nielsen 2014; Kiffner et al. 2015; Nina et al. 2017; Sack et al. 2018; Nthiwa et al. 2019). These pastoralist activities are also significant threats to wildlife, as human-assisted spread of pathogens to animals becomes the most probable certainty (Cunningham et al. 2017).

**Fig. 1:**
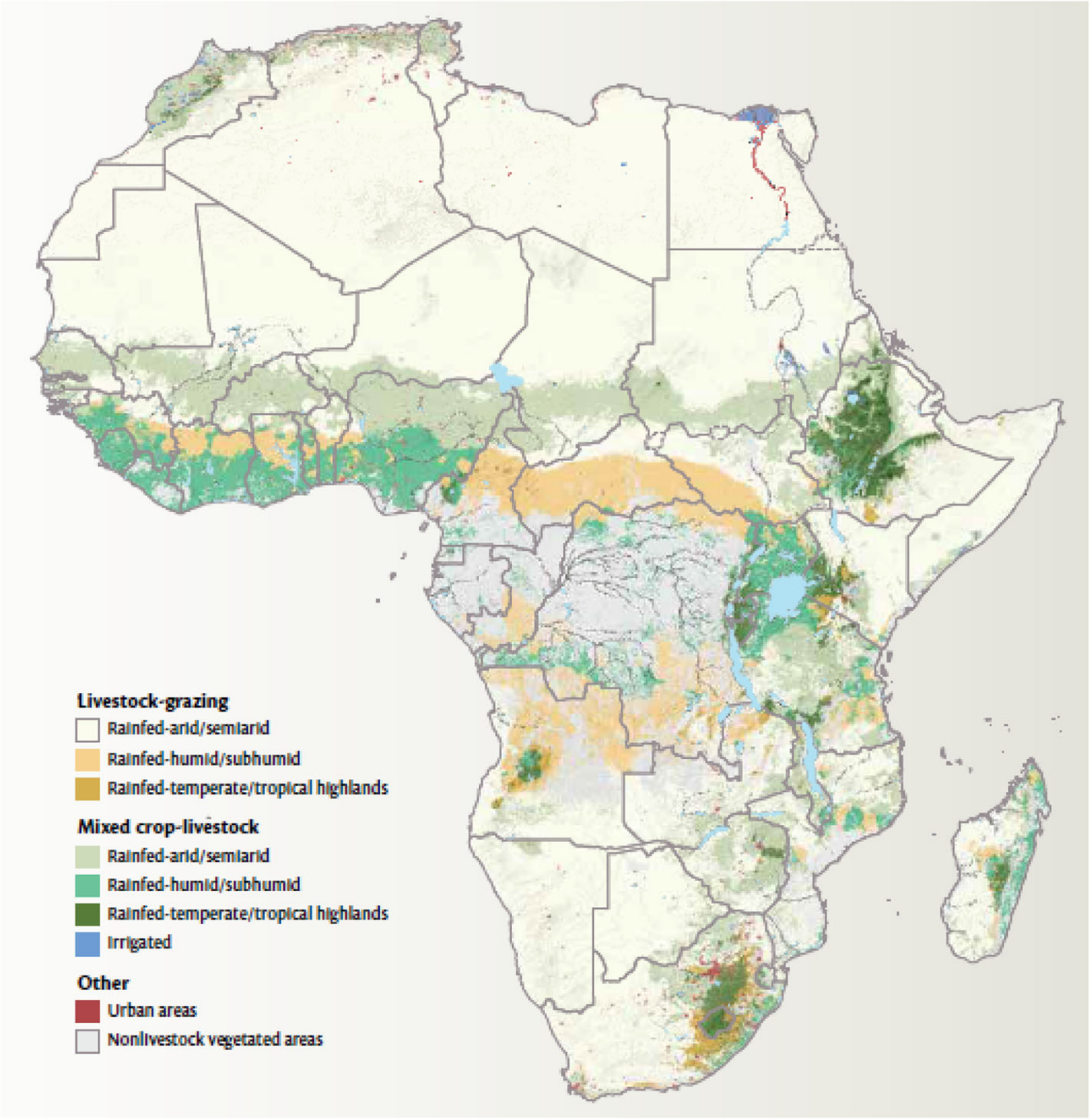
Livestock production systems by climate zone (Thornton 2014)

**Fig. 2:**
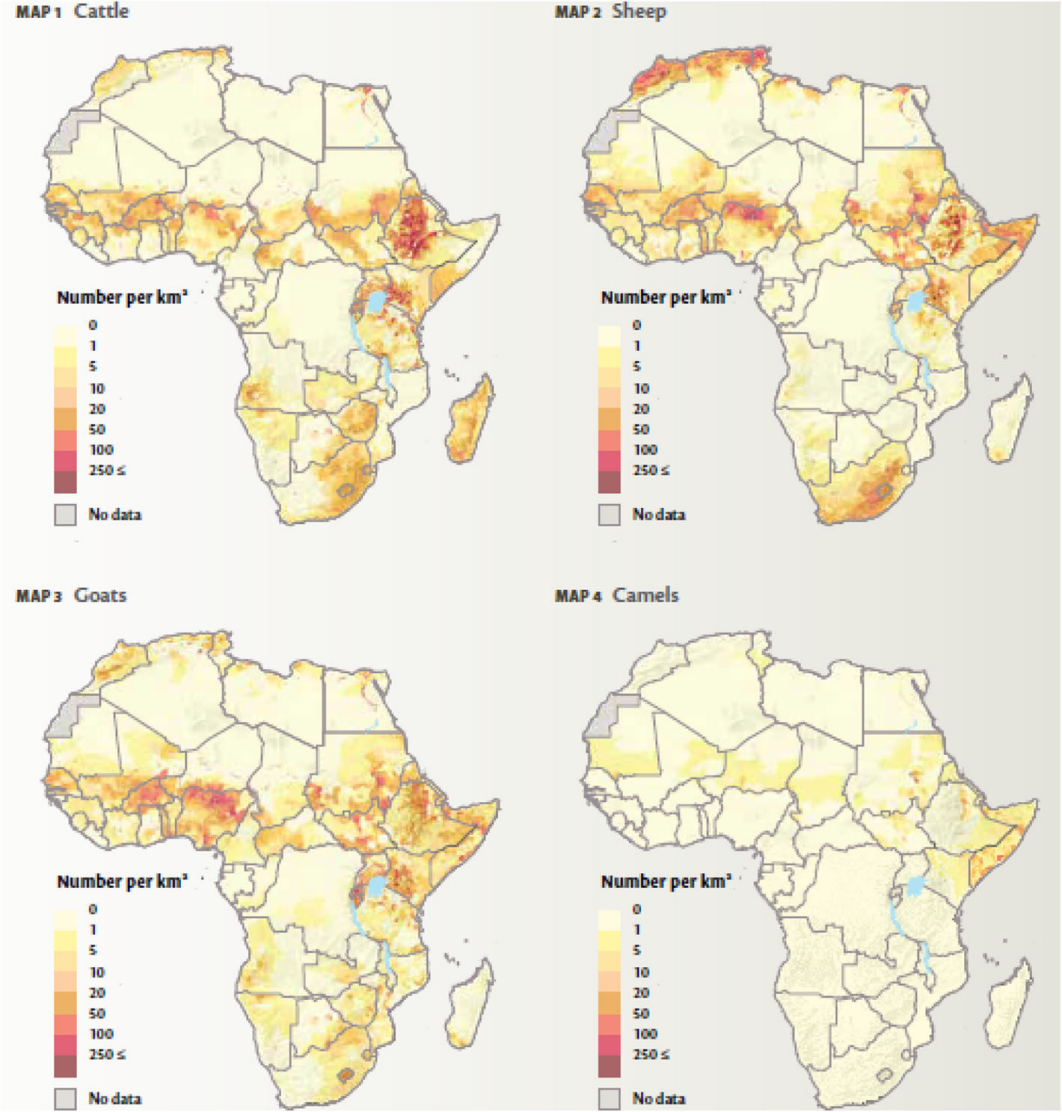
Distribution of ruminant livestock in Africa (Robinson et al. 2014)

Most of Africa’s livestock is produced within the pastoral systems occurring in the continent’s various dryland/semi-arid areas (Fig. 1). These areas produced the largest share of cattle, goats, sheep and camels in Africa (Fig. 2). Despite the difficulty in obtaining comprehensive livestock statistics and in particular livestock population numbers in Africa, earlier estimates had indicated that the livestock numbers have been growing. By 1999, the sub-Saharan Africa region had 191.3 million cattle, 158.7 million sheep, 182.1 million goats, 15.5 million pigs and 700 million chickens (Otte and Chilonda 2002). Countries such as Ethiopia, Sudan, Tanzania and Nigeria have 3.68%, 2.86%, 1.67% and 1.36% respectively share of the global average (Cook 2015). In terms of estimated numbers, this for example in the case of Sudan translates to 41.653 million cattle, 51.555 million sheep, 43.270 million goats, 4.521 million camels, 7.515 million donkeys and 784,000 horses, 90% of which are produced by pastoralists (Behnke 2012; Wilson 2018). Meanwhile, West Africa and the Sahel contain 25% of the cattle, 33% of sheep and 40% of goats in sub-Saharan Africa (Kamuanga et al. 2008).

Many pastoral communities recognise that interactions between animals and humans have the potential to transmit diseases either way. Bovine tuberculosis and Rift Valley fever have been documented in pastoral communities in Ngorongoro-Tanzania (Mangesho et al. 2017), Q-fever in Chad; *Mycobacterium bovis* infection among the Borana in Ethiopia and pastoralists in Ebonyi- south-eastern Nigeria (Greter et al. 2014; Adesokan et al. 2019); and anthrax outbreaks in Tanzania (Mwakapeje et al. 2018). Consumption of unpasteurised milk and being in direct contact with infected animal tissues like products of abortion and blood increase the risk of transmission of zoonotic diseases such as anthrax, bovine tuberculosis, brucellosis, cysticercosis, echinococcosis, rabies and zoonotic trypanosomiasis, equine encephalitis, hydatidosis/echinococcosis, leishmaniasis and rabies from wildlife to livestock and to humans (WHO 2009; Fevre et al. 2017; Muturi et al. 2018).

**Fig. 3:**
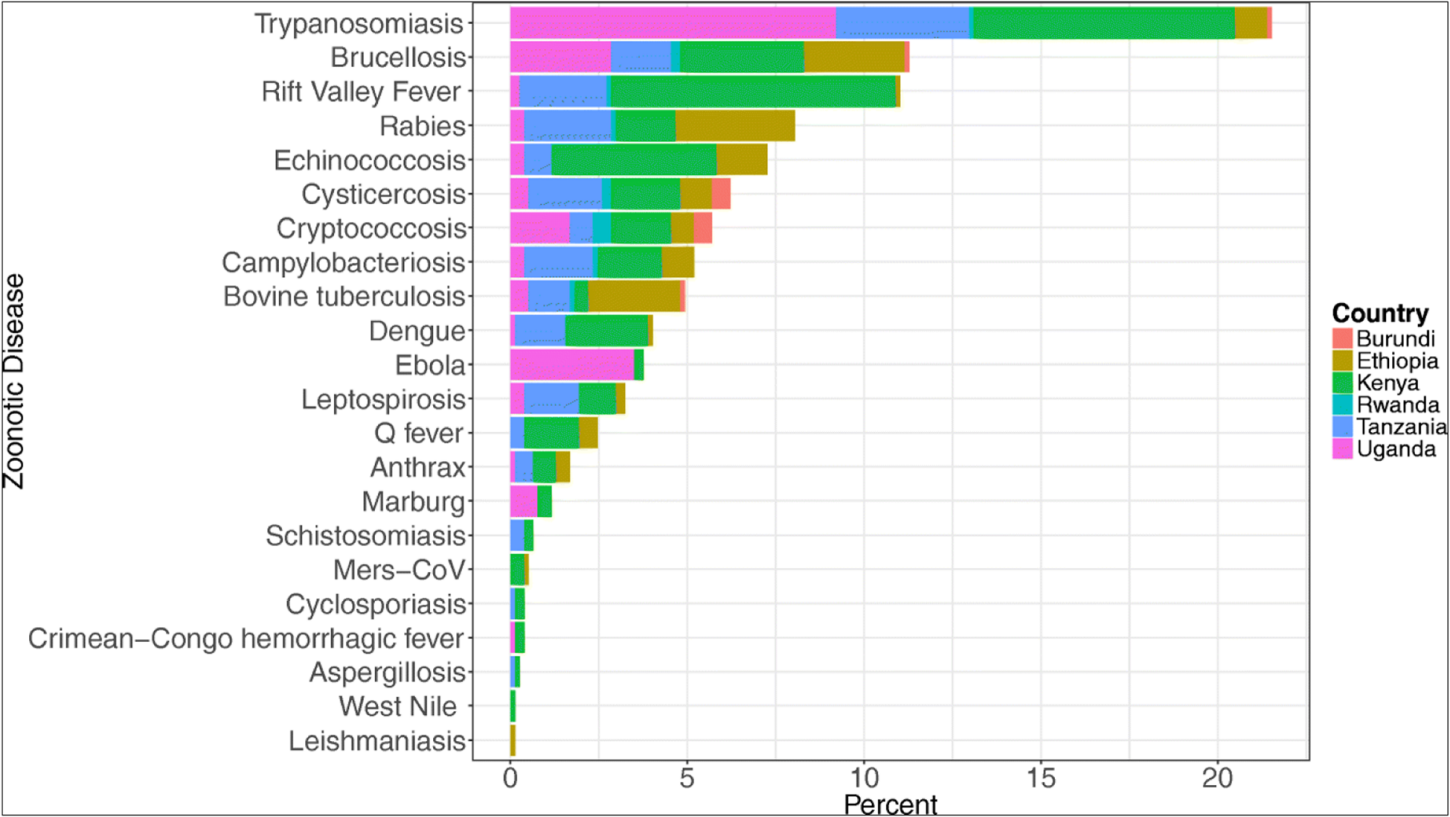
Twenty-two zoonotic diseases in East Africa by country (Source: Kemunto et al. 2018)

Major zoonotic infectious diseases which have devastating impacts on health, economy and livelihoods of African pastoralists include among others foot and mouth disease (FMD), Rift Valley fever (RVF) and Middle East respiratory syndrome coronavirus (MERS-CoV). There are also zoonotic infectious diseases that exist in the pastoral communities but largely remain neglected such as human African trypanosomiasis (HAT), brucellosis, cysticercosis/taeniasis, bovine tuberculosis and Q fever (Fevre et al. 2017; Elelu et al. 2019). Sub-Saharan Africa remains a hotspot region with a high prevalence of these zoonotic infectious diseases (Fig. 3), causing debilitating effects on people and their livestock (Kemunto et al. 2018). In addition, there are non-zoonotic infectious diseases such as peste des petits ruminants (PPR), contagious caprine pleuropneumonia (CCPP) and contagious bovine pleuropneumonia (CBPP) which further exacerbate livestock problems and losses among pastoralists in Africa.

Pastoralists by their way of life requiring close proximity with livestock, are presented with high exposure risks to zoonotic diseases. Pastoral communities and herds have a high prevalence of zoonotic infectious diseases and constitute a major part of the infectious disease burden in low-income countries (Asante et al. 2019). However, it is common to find pastoralists not recognising these zoonotic infectious diseases or attributing their cause to a different factor of nature, in spite of pastoralists’ renowned in-depth ethnoveterinary knowledge (Moritz et al. 2013). The current outbreak of severe acute respiratory syndrome coronavirus-2 (SARS-CoV-2) responsible for causing coronavirus disease-2019 (COVID-19) is reinvigorating the discourse on the disruptiveness of zoonotic infectious diseases globally. There are several coronaviruses of importance; some are zoonotic in nature while others are not. They are known as coronaviruses (CoV) because they belong to the ribonucleic acid (RNA) family of viruses that often have a characteristic crown (corona) of protein spikes around its lipid envelope (Wu et al. 2020a). Coronaviruses spill over, infecting humans and animals, while causing a range of effects including respiratory, gastrointestinal, hepatic and neurologic diseases among humans and animals alike (Wu et al. 2020b; Malta et al. 2020).

**Fig. 4:**
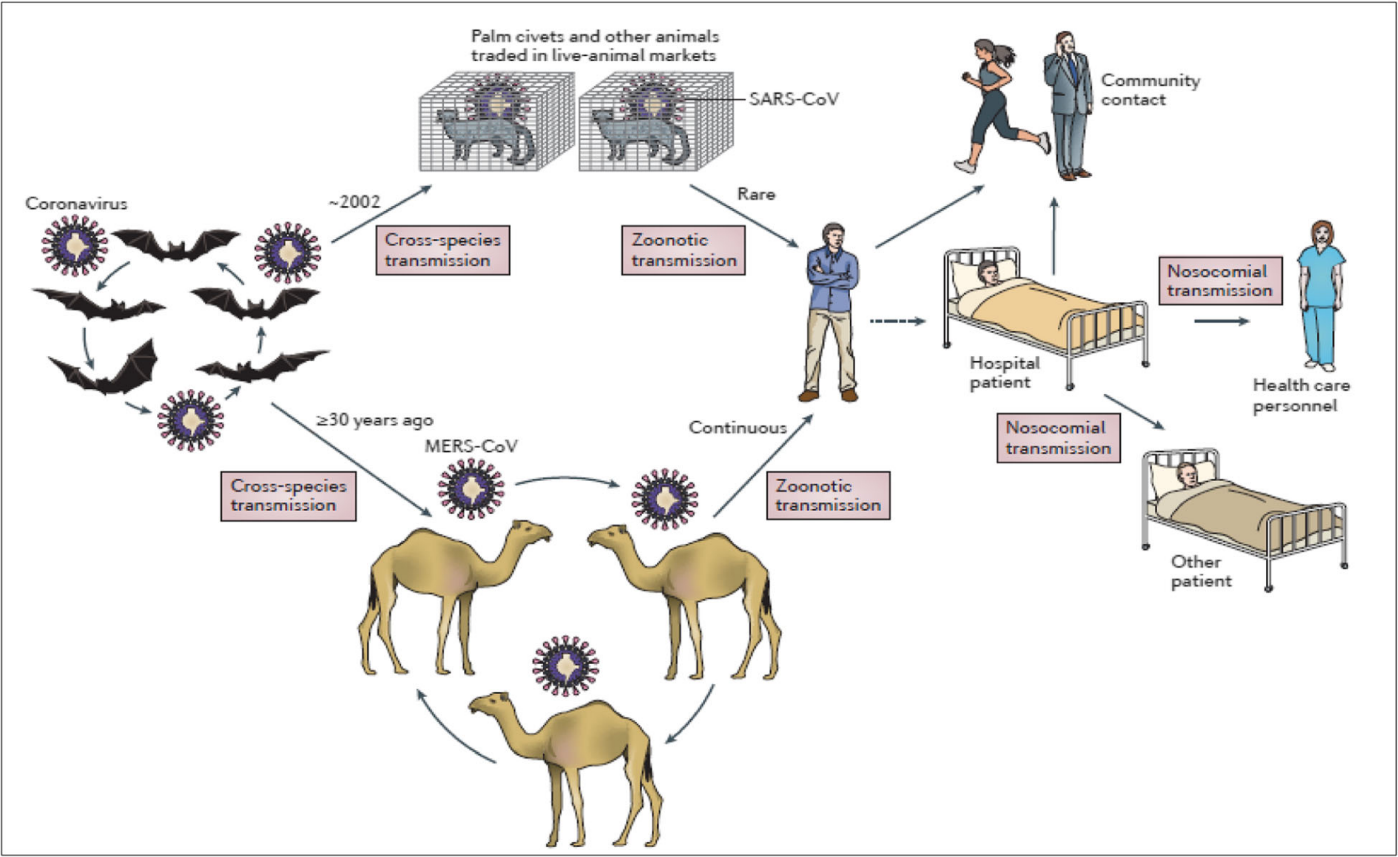
Emergence of SARS-CoV and MERS-CoV. Bats harbour a wide range of coronaviruses, including severe acute respiratory syndrome coronavirus (SARS-CoV)-like and Middle East respiratory syndrome coronavirus (MERS-CoV)-like viruses. SARS-CoV crossed the species barrier into masked palm civets and other animals in live-animal markets and genetic analysis suggests that this occurred in late 2002. Several people in close proximity to palm civets became infected with SARS-CoV. A MERS-CoV ancestral virus crossed the species barrier into dromedary camels; serological evidence suggests that this happened more than 30 years ago. Abundant circulation of MERS-CoV in dromedary camels results in frequent zoonotic transmission of this virus. SARS-CoV and MERS-CoV spread between humans mainly through nosocomial (hospital-acquired) transmission (Source de Wit et al. 2016)

The current outbreak of coronaviruses is not a new phenomenon as there have been previous outbreaks. Seven coronaviruses (229E, NL63, OC43, HKU1, MERS-CoV, SARS-CoV) that infect humans have been identified since the 1960s (Woo et al. 2012; Wang et al. 2020). Recently, two outbreaks of coronaviruses have been recorded with devastating effects, these being the Middle East respiratory syndrome coronavirus (MERS)-CoV and severe acute respiratory syndrome coronavirus (SARS)-CoV (Wang et al. 2020). Studies have shown that SARS-CoV was transmitted from either bats or civets to humans while MERS-CoV from dromedary camels to humans (Fig. 4, de Wit et al. 2016; Dutton 2020; NIAID 2020). While the rest of the world’s attention is currently geared towards the COVID-19 pandemic, there is equally a re-emergence of MERS-CoV in Saudi Arabia. From 1 to 29 February 2020, there have been 18 cases of MERS-CoV infection with at least five deaths (WHO 2020). Previous outbreaks of MERS-CoV since 2012 have led at least to 2538 infectious with 871 deaths. Serological evidence available so far indicates that there has been a circulation of MERS coronavirus (MERS-CoV) among dromedary camels in the greater Horn of Africa (Somalia and Sudan) as far back as 1983 and in Saudi Arabia as far back as 1992. Further, MERS-CoV has also been identified in Nigerian camels, but this trait is genetically different from that found in camels and humans in the Middle East (Younan et al. 2016).

As the camel trade between the Horn of Africa and the Middle East rises, in 2015, the HoA exported 5.3 million animals to the Gulf region, the highest in two decades (Patinkin and Dahir 2016), the interaction and pathogen flows are likely to increase between the two regions. This raises the level of risk in pastoral Africa particularly because the prevalence of MERS-CoV in Saudi Arabian camels has been shown to be higher than those from Africa. Further, intra-Africa trade for camels from the Sahelian belt countries to North Africa is also common (Napp et al. 2018); this could also further increase the spread of MERS-CoV and other infectious diseases in that part of the continent. It is also important to note that the case fatalities in MERS-CoV are particularly high and as such should not be taken lightly (de Wit et al. 2016).

Recent reports about the emergence of infectious diseases in most of sub-Saharan Africa are linked to zoonotic infectious diseases. The frequency and trend of the emergence and re-emergence of diseases such as Ebola, Marburg, Rift Valley fever and anthrax appear to be on the rise (Kemunto et al. 2018). Several risk factors catalyse the transmissibility of zoonotic infectious diseases, including livestock slaughter, handling and preparation of animal-origin food and consumption of such animal products, especially when improperly cooked or in their raw form (Mangesho et al. 2017). Because pastoralists are in daily contact with livestock as well as with wildlife, they remain highly exposed to zoonotic-oriented pathogens. Further, as pastoral communities are one of the key sources of livestock and livestock products, especially beef, to most rural and urban communities in sub-Saharan Africa, they potentially serve as an important source of transmission to other communities. This risk further rises among transhumant pastoralists as they traverse various regions and across borders in search of pasture and water for their livestock.

Current globalisation trends are shaping and reshaping the world in a unique manner. There is no longer a place so divorced from the rest of the world. Global trade interdependence, travel, migration and international markets have all become defining factors in shaping the course of infectious disease dynamics: rise, emergence and re-emergence across the world (Mackey and Liang 2012).

Most of the affected persons during outbreaks of these zoonotic infectious diseases in Africa have been from resource-poor communities, isolated and with very marginal public health care services (Cascio et al. 2011; Smith et al. 2019). African governments, with international support, have over the years attempted to control these infectious diseases. For example, the Government of Tanzania had by 1969 eliminated rinderpest and contagious bovine pleuropneumonia (CBPP) and had managed to bring tick-borne diseases under control. However, re-emergence of rinderpest in Tanzania in the 1980s and CBPP in the 1990s has persisted to date, further undermining the successes achieved in earlier years (Rweyemamu et al. 2006). This underscores that in the case of zoonotic infectious diseases, attention has tended to focus more on the emerging zoonoses that pose global economic and health threats, but with minimal attention paid to the endemic zoonotic diseases (Kemunto et al. 2018). This has particularly been well-demonstrated in the current global pandemic of novel coronavirus (COVID-19) that has drawn the attention of the global community.

In this review paper, we stress the issue of emerging zoonotic infectious diseases over Africa, especially pastoral Africa, and point out the need for a coordinated effort to collaboratively respond to the challenges that these infectious diseases pose. We in particular propose that the One Health initiative offers an umbrella for coordinated action towards addressing some emerging and re-emerging zoonotic infectious diseases as well as endemic zoonotic diseases affecting pastoral communities in Africa.

## One Health approach in the context of emerging and re-emerging zoonotic diseases

Because zoonotic infectious diseases, especially emerging infectious diseases such as COVID-19, SARS-CoV and MERS-CoV, are influenced by a number of interacting factors and have tended to rapidly spiral in infections, they ought to be addressed dynamically by diverse actors and sectors of society including veterinary, public health, medicine, environmental science, food safety, economics and public policy among others (Mackey et al. 2014; Tappero et al. 2017). This calls for a deployment of unique approaches able to provide a trusted platform for organising diverse stakeholders into a common resource of value. One such approach that has become highly considered is the One Health approach that engages interdisciplinary and multi-stakeholder participation locally, nationally and globally in areas of human and animal health, agriculture and the environment (Kaplan et al. 2009). Already, a recent assessment of Africa’s preparedness and vulnerability to COVID-19 has shown very high risk amidst low capacity, which implies high vulnerability levels for most of the countries assessed (Gilbert et al. 2020).

One Health is underpinned by recognition of the interdependence of human health, animal health and environmental health. It seeks to achieve better public health outcomes through the understanding and prevention of risks that originate at the interface of humans, animals and their environments. The One Health approach has appeared as a unique example of alternative approaches that is able to aggregate and align various efforts and facilitate them to work synergistically and effectively.

Most of the necessary players in the network exist but work in isolation. For example, veterinarians are not connected with occupational human physicians, and in turn, they are not in contact with general doctors who are at the forefront of the disease treating humans. Ecosystem experts and disease ecologists who often analyse interfaces between wildlife, livestock and humans are most times not in touch with epidemiologists. Accordingly, the current system and network that is in position to receive and act on early warnings at different levels is missing, and where it exists, it is arguably weak and not glued together (EClinicalMedicine 2020). Yet, ecologists, epidemiologists, policy-makers, behavioural specialists and other experts need to work together to understand the multifaceted connections among potential host species to identify risk factors for disease transmission and ascertain efficient management actions.

One Health application is gaining traction in Africa (Rwego et al. 2016). One Health approach strategically responded to endemic Q fever in Chad through joint animal and human vaccination interventions. This led to increased access to primary health care services among pastoralists. Similarly, cases of *Mycobacterium bovis* among the Borana pastoralists in Ethiopia decreased with the application of One Health in the intervention programmes (Greter et al. 2014). Among the Ethiopian Somali pastoralists, One Health approach was instrumental in the control of bovine tuberculosis that was rampant in the area, control owing to close interaction between people, animals and their environment, because of their nomadic pastoral production practices (Mohamed 2020). Further, several countries—Chad, Kenya, Ethiopia, Tanzania, Uganda, Cameroon and Mali—are translating the One Health approach into reality through advancing institutional development as well as guiding programme development. For example, Cameroon created the National Zoonoses Program for emerging and re-emerging infectious diseases (Rwego et al. 2016); Kenya established an inter-ministerial zoonotic disease unit; the science of One Health was integrated at Tanzania’s Nelson Mandela African Institute of Science and Technology (NMAIST), while a diagnostic laboratory sharing in Mali was instituted (Kamani et al. 2015). These interventions recognise that zoonotic infectious disease management depends on active collaboration of multiple stakeholders at various levels; this is necessary to increase surveillance capacity, response rates and monitoring of the human health and/or livestock populations and appraisal of related disease risks (Valeix 2018). Experts and stakeholders involved in One Health in Africa recognise technical capacity limitations, and as such, three regional networks have been created: Afrique One, Southern African Centre for Infectious Disease Surveillance (SACIDS) and One Health Central and Eastern Africa (OHCEA) are all championing capacity-building in Africa (Rwego et al. 2016).

**Fig. 5:**
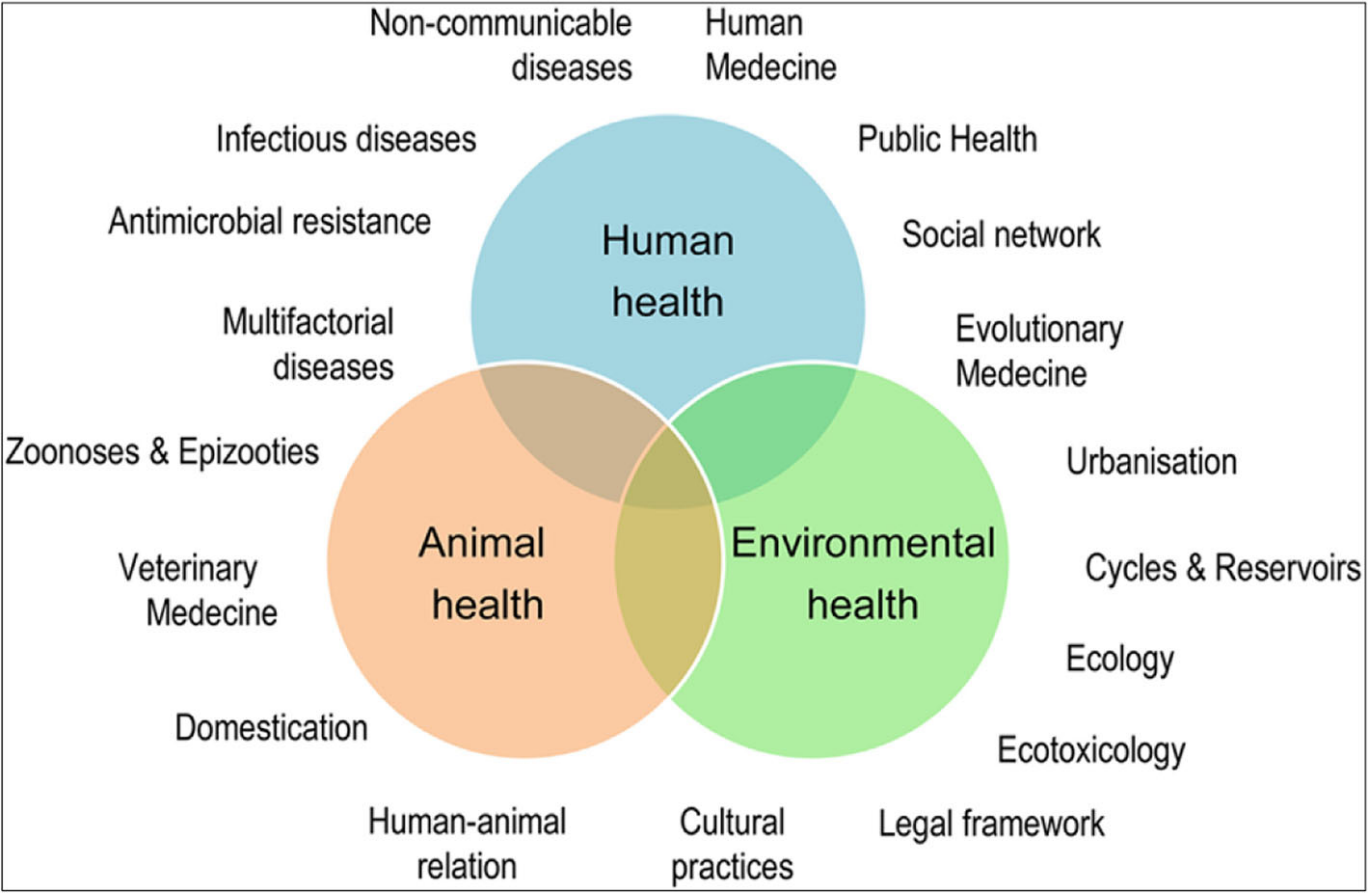
One Health concept: holistic, transdisciplinary and multi-sectoral approach to Health (Source: Destoumieux-Garzón et al. 2018)

As the novel coronavirus outbreak has been greatly linked to Wuhan patients that had visited the wet fish and wild animal market that sold live animals such as poultry, bats, marmots and snakes (Lu et al. 2020), it becomes vital to take a critical look into the human–animal–ecosystem interaction in the evolution and emergence of pathogens. The One Health approach provides this framework for action that is holistic and transdisciplinary (Fig. 5; Destoumieux-Garzón et al. 2018).

As pointed by Pigott et al. (2017), there is a strong heterogeneity of occurrence, re-emergence and spillover of zoonotic originating infectious diseases across Africa. Most of these are increasing with ecosystem and habitat degradation and unhealthy human–wildlife interactions (Omoleke et al. 2016; Fenollar and Mediannikov 2018) as humans get into contact with disease reservoirs emerging from fragmented and disturbed habitats (Rulli et al. 2017). While COVID-19 has been imported into Africa, its transmission has followed a similar conceptualised pattern of viral zoonotic progression from animal reservoir to global pandemic. Accordingly, it is critical for public health practitioners to incorporate environmental considerations, including ecology and evolutionary aspects, within One Health approach for an innovative and effective control of zoonotic infectious diseases.

Despite One Health gaining traction, its implementation has still lagged behind. This has been attributed to differences in perspectives among the various professions, experts and actors, while social dimensions and power dynamics have been indicated as some of the factors for this limited translation at local and national levels (Valeix 2018). Thus, building consensus among the diverse experts remains one of the outstanding challenges that One Health has to surmount not only in Africa but also globally. In the case of Africa, this will particularly become more complex owing to the breadth and dependence on social safety nets often provided by the state, requiring significant investments amidst competing state priorities, and local actions within the food habits of the people, where wildlife for example is part of the protein sources. Further, as pointed out by Gilbert et al. (2020), Africa’s vulnerability to disease risk is varied. In this regard, despite facing the same threat of emerging infectious diseases such as COVID-19, the variation in risk across the continent influences how experts, policy and decision leaders respond, for example with regard to investment in public health and the strategies for intervention. Growing the One Health approach within the continent and in particular at local and national levels requires capacity-building in the first place. As an integrative and transdisciplinary practice, to move experts, practitioners and policy leaders to work together is no simple task. Deliberate effort and investment are required. In addition, planning and policy processes ought to be well-streamlined rather than reactive and merely responding to outbreaks of epidemics and pandemics. Thus, there is an appeal for One Health to address endemic and neglected diseases that are now barely addressed under global considerations (Okello et al. 2014).

## Conclusions

Pastoral communities across Africa live in close proximity to livestock, depend heavily on their livestock and livestock products and are one way or the other attuned to wildlife as either domestic souvenirs or consumed as food. These actions are key predisposing agents and factors that increase their susceptibility to zoonotic infectious diseases. We have also seen that pastoralists may likely become spatial and temporal transmitters of emerging and re-emerging infectious diseases across Africa, owing to their supply of livestock and livestock products, especially meat, and their transhumant livestock management style. Moreover, as the world has now focused primarily on the outbreak of COVID-19, the outbreak of equally dangerous coronavirus-associated disease (MERS-CoV) that has already killed five people in Saudi Arabia is barely reported anywhere. This outbreak is important to the pastoral communities in Africa because of increasing camel trade from Somalia, Ethiopia, Sudan, Eritrea and Djibouti to the Gulf States especially Yemen and Saudi Arabia.

There are complexities in the transmissibility of zoonotic infectious diseases, the global spread, disruptiveness to economies and emergency situations that they portend. One Health offers a strong approach to bring together diverse actors and sectors of society to act together towards prevention, response, mitigation and monitoring of zoonotic infectious diseases. The One Health approach at the same time can address challenging issues of ecosystem degradation that is increasing human–wildlife contact, thereby increasing spillover risks. To implement One Health means securing political and policy support in addressing long-term requisite investments and policies.

## Data Availability

All reviewed and cited articles have been referenced.

## Abbreviations

CBPP: Contagious bovine pleuropneumonia
CCPP: Contagious caprine pleuropneumonia
COVID-19: Coronavirus disease-2019
FMD: Foot and mouth disease
HAT: Human African trypanosomiasis
MERS-CoV: Middle East respiratory syndrome coronavirus
NIAID: National Institute of Allergy and Infectious Diseases
PPR: Peste des petits ruminants
NA: Ribonucleic acid
RVF: Rift Valley fever
SARS-CoV-2: Severe acute respiratory syndrome coronavirus-2
WHO: World Health Organization
